# Necrotizing Fasciitis Among Patients With Liver Cirrhosis in Texas, 2001 - 2010: A Population-Based Cohort Study

**DOI:** 10.14740/jocmr2420w

**Published:** 2015-12-28

**Authors:** Lavi Oud, Phillip Watkins

**Affiliations:** a Division of Pulmonary and Critical Care Medicine, Department of Internal Medicine, Texas Tech University Health Sciences Center at the Permian Basin, 701 W. 5th St., Odessa, TX 79763, USA; b Clinical Research Institute, Texas Tech University HSC, 3601 4th Street, MS6238, Lubbock, TX 79430, USA

**Keywords:** Mortality, Organ failure, Necrotizing fasciitis, Cirrhosis, Resource utilization

## Abstract

**Background:**

Liver cirrhosis is a risk factor for necrotizing fasciitis (NF), and is associated with markedly worse outcomes than for NF among non-cirrhosis patients. Only limited, mostly single-center, data were reported to date on the epidemiology, clinical features, resource utilization and outcomes of NF among patients with cirrhosis.

**Methods:**

We studied a population-based cohort of adult hospitalizations associated with cirrhosis, who had a diagnosis of NF during the years 2001 - 2010, using the Texas Inpatient Public Use Data File. The annual volume of NF hospitalizations was benchmarked against all annual hospitalizations with a diagnosis of cirrhosis. The patterns of demographics, chronic comorbidities, evolving organ failure, resource utilization and outcomes were examined.

**Results:**

There were 371,745 hospitalizations associated with liver cirrhosis, with 381 NF hospitalizations during study period. The annual volume of NF hospitalizations rose 7.9%/year (P = 0.0287), while its incidence among cirrhosis-associated hospitalizations remained unchanged (P = 0.2955). Non-cirrhosis comorbidities were reported in 69.6% and ICU care was required in 67.2% of NF hospitalization. The key changes noted between 2001 - 2003 and 2008 - 2010 among NF hospitalizations included rising mean (SD) Deyo-Charlson index 2.4 (1.5) vs. 3.9 (2.4) (P < 0.0001), development of ≥ 3 organ failures in 9.1% vs. 39.8% (P < 0.0001), and discharge to long-term care facilities 7.8% vs. 21.1% (P = 0.0204). Hospital mortality was unchanged (26% vs. 33.1%; P = 0.3659). Inflation-adjusted total hospital charges did not change (P = 0.1025) during study period.

**Conclusions:**

The present cohort of NF associated with liver cirrhosis is the largest reported to date. A rising annual volume of NF events matched a corresponding increase in cirrhosis-associated hospitalizations. There was increasing burden of chronic comorbidity and rising severity of illness, with a majority of patients requiring ICU care. Case fatality was high and there has been increasing residual morbidity among hospital survivors. The observed findings warrant further study in other populations.

## Introduction

Cirrhosis is associated with increased risk of infection, resultant sepsis and sepsis-associated mortality [1] due to multiple immunological and anatomical defects [2, 3]. Indeed, some investigators have suggested that liver cirrhosis is the most common form of acquired immunodeficiency [3]. Skin and soft tissue infection account for a minority of infectious complications of cirrhosis [4], but necrotizing infections can be associated with especially high morbidity and mortality.

Necrotizing fasciitis (NF) is a rare soft tissue infection manifesting as necrosis of subcutaneous tissues and fascia. NF commonly results in severe illness with high resource utilization, with previously reported case fatality exceeding 40% in single-center studies [5] in the general population. However, more recent population-level reports described case fatality around 5-12% [6-8].

Cirrhosis is considered a risk factor for NF [9] and is considered a risk factor of increased mortality among NF patients [10, 11]. However, NF among cirrhotic patients has not been adequately characterized and has been described for the most part only in case reports [12-14] and small single-center cohorts, predominantly from Taiwan [15, 16]. Because of the rarity of NF among patients with cirrhosis, longitudinal studies trends of its epidemiology, clinical features, and outcomes are not readily amenable to traditional clinical investigation. While local studies can provide wealth of clinical information, population-level investigations of administrative data sets, though lacking details of clinical manifestations, time course to events, and proper anatomical detail, can provide broader data on longitudinal patterns of the aforementioned domains with selected clinical characteristics and resource utilization. The latter data can inform focused clinical studies and, subsequently, clinical practice. No cohort studies on NF among patients with cirrhosis in the United States (US) have been reported, to our knowledge.

We thus sought to examine population-level longitudinal patterns of the epidemiology, clinical features, resource utilization, and outcomes of NF among patients with cirrhosis in Texas.

## Material and Methods

### Data sources

We used the Texas Inpatient Public Use Data File (TIPUDF), a longitudinal data set maintained by the Texas Department of State Health Services [17], to perform a retrospective, population-based cohort study of NF among hospitalized patients with a diagnosis of cirrhosis in the state during 2001 - 2010. The data set includes detailed de-identified inpatient discharge data from all state-licensed hospitals, with the exception of those exempt by state statute from reporting to the Texas Health Care Information Collection. The facilities included in the mandated report account for 93-97% of all hospital discharges. The TIPUDF data set includes demographic, clinical, resource utilization, and outcome information. The data set includes up to 25 discharge diagnoses, and up to 25 procedures, coded using the International Classification of Diseases, Ninth Revision, Clinical Modification (ICD-9-CM). Patients’ gender is masked among those with diagnoses of infection with the human immunodeficiency virus, or with drug or alcohol abuse. Because we used a publicly available, de-identified data set, this study was determined to be exempt from formal review by the Texas Tech Health Sciences Center Institutional Review Board.

### Study population

We used ICD-9-CM codes 571.2, 571.5, and 571.6 to identify hospitalizations of Texas residents 18 years or older with a primary or secondary diagnosis of cirrhosis (termed cirrhosis-associated hospitalizations in the remainder of manuscript) between 2001 and 2010. We then used an ICD-9-CM code 728.86 to identify those hospitaIizations among the former group with a primary or secondary diagnosis of NF. The latter group formed the primary cohort for the present study.

### Data collection

We collected data on patients’ age, gender, race/ethnicity (categorized as non-Hispanic black (black), non-Hispanic white (white), Hispanic, and other), health insurance (categorized as private, Medicare, Medicaid, uninsured, and other), chronic co-morbid conditions (based on the Deyo-Charlson index [18]), obesity, smoking, drug and alcohol abuse, other sites of infection (Supplementary Table 1, www.jocmr.org), type and number of failing organs (OFs) (Supplementary Table 2, www.jocmr.org), admission to an ICU (defined as presence of an intensive care unit charge greater than $0), life-support interventions (mechanical ventilation, central venous catheterization, and hemodialysis) (Supplementary Table 3, www.jocmr.org), total hospital charges, hospital length of stay, and disposition at the end of hospitalization. Severity of illness was based on the number of OFs [19], as modeled by the coding system reported by Lagu et al [20]. Although selected microbiology data are included in administrative data sets, they are not reported in the majority of hospitalizations of infected patients [21]. In addition, other sites of infection are commonly reported among NF patients [8, 22], and administrative data sets do not provide information of the source of reported microbiological data. The aforementioned constraints can lead to skewed information with inappropriate internal and external validity. We thus elected to not report microbiology information in the present cohort.

### Outcomes

Examined outcomes included incidence of NF among cirrhosis-associated hospitalizations, non-cirrhosis chronic comorbidity, number and type of OF, resource utilization, hospital mortality, and disposition among hospital survivors.

### Data analysis

Because there is lack of reliable data on the annual prevalence of liver cirrhosis in Texas, we have benchmarked the annual volume of NF hospitalizations against that of all cirrhosis-associated hospitalizations in a given year, as surrogate measure of NF incidence in this population.

The mortality associated with NF was examined as case fatality (defined as the number of NF hospitalizations who died in the hospital, divided by the total number of NF hospitalizations for an examined group).

Group data are reported as numbers (percentages) for categorical variables and mean (standard deviation (SD)) or median (interquartile range (IQR)) for continuous variables, as appropriate. Because commonly skewed data on number of OFs, length of stay, and hospital charges are often reported as mean (SD), we included both median (IQR) and mean (SD) data on these domains to allow comparison to prior studies. Distribution of normality was examined by Kolmogorov-Smirnov test. Categorical data were compared by two-sided Chi-square test. Mann-Whitney U test and *t*-test were used to compare continuous data, as appropriate. Log-transformed data were used to perform linear regression analyses to explore the annual trends of NF volume, incidence of NF among cirrhosis-associated hospitalizations, hospital length of stay, and total hospital charges. When examining changes of key characteristics at the start vs. end of past decade, we used combined 3-year data to enhance precision of comparisons. Total hospital charges were examined using inflation-adjusted (2010) dollars [23]. All statistical analyses were performed using MedCalc version 15.6 (MedCalc Software, Ostend, Belgium) and SAS version 9.3 (SAS Institute, Cary, NC, USA). A two-sided P value < 0.05 was considered statistically significant.

## Results

There were 371,745 hospitalizations with a diagnosis of liver cirrhosis, and 381 NF hospitalizations among the latter during the 2001 - 2010 period. Most (85.3%) NF hospitalizations were younger than 65 years of age. Hispanics constituted the largest group (44.1%) of NF hospitalizations and 62.6% were males (among hospitalizations with reported gender). Private insurance was the most common type of health insurance (29.4%), while 21.8% of NF hospitalizations lacked health insurance. Non-cirrhosis chronic comorbidities were reported in 265 (69.6%) NF hospitalizations, with diabetes mellitus (31.2%), and chronic kidney disease (20.7%) being the most common. The demographics, health insurance, and chronic comorbidities of NF hospitalizations are detailed in Table 1. Other (non-NF) sites of infection were reported in 115 (30.2%) NF hospitalizations, with the most common reported sites being the urinary tract (14.2%), intra-abdominal (10.5%), and respiratory (6.3%).

**Table 1:** Characteristics of NF Hospitalizations With a Diagnosis of Cirrhosis

Group	n = 381
Age (years), n, (%)	
18 - 64	325 (85.3)
≥ 65	56 (14.7)
Gender, n, (%)^a^	
Female	89 (37.4)
Male	149 (62.6)
Race/ethnicity, n (%)	
Hispanic	168 (44.1)
Black	26 (6.8)
White	162 (42.5)
Other	24 (6.3)
Missing	1 (0.3)
Health insurance, n (%)	
Private	112 (29.4)
Medicare	106 (27.8)
Medicaid	65 (17.1)
Uninsured	83 (21.8)
Other	14 (3.7)
Missing	1 (0.3)
Chronic comorbidities, n, (%)^b^	
Any^c^	265 (69.6)
Congestive heart failure	40 (10.5)
Peripheral vascular disease	35 (9.2)
Chronic pulmonary disease	36 (9.4)
Diabetes mellitus	119 (31.2)
Connective tissue disease	4 (1)
Cerebrovascular disease	9 (2.4)
Chronic kidney disease	79 (20.7)
Malignancy	16 (4.2)
HIV infection	5 (1.3)
Deyo-Charlson score	
Median (IQR)	3 (1 - 4)
Mean (SD)	3.2 (2.2)
Obesity, n (%)	31 (8.1)
Drug abuse, n (%)	28 (7.3)
Smoking, n (%)	45 (11.8)
Alcohol abuse, n (%)	132 (34.6)

^a^Gender was masked in 143 hospitalizations. The denominator used to derive female/male percentage for the cohort was based on hospitalizations with available gender designation (n = 238). ^b^Based on the Deyo-Charlson comorbidity index. ^c^Excluding liver cirrhosis. HIV: human immunodeficiency virus; IQR: interquartile range; SD: standard deviation.

The annual volume of NF hospitalizations with cirrhosis rose 7.9%/year (P = 0.0287). However, when benchmarked against the annual hospitalizations with a diagnosis of cirrhosis, the overall incidence of NF hospitalizations was 10.2 per 10,000 cirrhosis hospitalizations/years, with no significant change over study period (P = 0.2955).

One or more organ failures were reported in 73% of NF hospitalizations, involving most commonly the hematological (42.5%), renal (35.7%) and respiratory (33.3%) systems. An amputation was required in 10 NF hospitalizations (2.6%). One or more examined life-support interventions were used in 188 (49.3%) NF hospitalizations. The key changes of the examined clinical characteristics, resource utilization, and outcomes among NF hospitalizations between 2001 - 2003 and 2008 - 2010 are outlined in Table 2. The mean (SD) Deyo-Charlson comorbidity index rose from 2.4 (1.5) to 3.9 (2.4) (P < 0.0001). NF hospitalizations with one or more organ failure increased from 43 (55.8%) to 113 (85%) during past decade (P < 0.0001). Use of life-support interventions rose significantly for mechanical ventilation and hemodialysis and tended to rise for central venous catheterization during study period, but did not reach statistical significance. There was no significant change in use of the examined interventions among NF hospitalizations with specific failing organs between 2001 - 2003 and 2008 - 2010: 1) mechanical ventilation: 64.7% vs. 72% (P = 0.7941) among those with respiratory failure; 2) central venous catheterization: 33.3% vs. 49.1% (P = 0.5054) among those with cardiovascular failure; 3) hemodialysis: 15.4% vs. 23.4% (P = 0.7860) among those with acute renal failure. Hyperbaric oxygen therapy was used in nine NF hospitalizations (2.4%).

**Table 2:** Changes in the Clinical Characteristics, Resource Utilization, and Outcomes of NF Hospitalizations With a Diagnosis of Cirrhosis

Variable	2001 - 2003 (n = 77)	2008 - 2010 (n = 133)	P
Deyo-Charlson score			
Median (IQR)	2 (1 - 3.3)	4 (2 - 5.3)	< 0.0001
Mean (SD)	2.4 (1.5)	3.9 (2.4)	
Number of failing organs			
Any, n (%)	43 (55.8)	113 (85)	< 0.0001
Median (IQR)	0 (0 - 1.3)	2 (1 - 4)	< 0.0001
≥ 3 organ failures, n (%)	7 (9.1)	53 (39.8)	< 0.0001
Organ failures, n (%)			
Respiratory	17 (22.1)	50 (37.6)	0.0299
Cardiovascular	12 (15.6)	53 (39.8)	0.0004
Renal	13 (16.9)	64 (48.1)	< 0.0001
Hepatic	3 (3.9)	25 (18.8)	0.0044
Hematological	22 (28.6)	72 (54.1)	0.0006
Metabolic	5 (6.5)	26 (19.5)	0.0179
Neurological	0 (0)	13 (9.8)	0.0112
Life-support interventions, n (%)			
Mechanical ventilation	11 (14.3)	36 (27.1)	0.0489
Central venous catheterization	18 (23.4)	48 (36.1)	0.0787
Hemodialysis	2 (2.6)	17 (12.8)	0.0258
Hospital length of stay, days			
Median (IQR)	13 (8 - 26)	11 (5 - 19.3)	0.1005
Mean (SD)	18.6 (16.7)	15.9 (14.9)	
Total hospital charges, dollars^a^			
Median (IQR)	80,805 (45,455 - 159,356)	101,678 (46,482 - 158,857)	0.5026
Mean (SD)	137,135 (200,766)	142,023 (174,999)	
Patient disposition, n (%)			
Hospital mortality	20 (26)	44 (33.1)	0.3569
Home	27 (35.1)	51 (38.3)	0.7444
Short-term facility	17 (22.1)	4 (3)	< 0.0001
Long-term facility	6 (7.8)	28 (21.1)	0.0204
Other^b^	6 (7.8)	5 (3.8)	0.3458
Missing	1 (1.3)	1 (0.8)	0.7308

^a^Hospital charges are adjusted for inflation to 2010 dollars. ^b^Left against medical advice, hospice. IQR: interquartile range; SD: standard deviation.

Admission to an intensive care unit (ICU) was required in 256 (67.2%) of NF hospitalizations. The mean (SD) hospital length of stay was 17.9 (17.2), with mean (SD) inflation-adjusted total hospital charges $144,820 (171,223). There was no significant change in annual hospital length of stay (P = 0.2566) and inflation-adjusted total hospital charges (P = 0.1025) among NF hospitalizations.

One hundred and seventeen patients (30.7%) died during hospitalization, with a non-significant upward trend over the past decade. The rate of discharge to a long-term facility increased from 7.8% to 21.1% between 2001 - 2003 and 2008 - 2010 (P = 0.0204). Only 142 (37.3%) NF hospitalizations were discharged home during study period.

## Discussion

The key findings of the present study include progressive rise in the annual volume of NF hospitalizations among patients with liver cirrhosis. However, the observed growth matched the corresponding rise in hospitalization needs among cirrhotic adults in the state. NF hospitalizations had common and increasing burden of non-cirrhosis chronic illness, and showed increased severity of illness over time. Over two in three NF hospitalizations in our cohort required care in an ICU, and required prolonged hospitalization with high hospital charges. Case fatality was high and hospital survivors sustained persistent morbidity with only about one in three discharged home.

The present study is, to our knowledge, the first population-level examination of NF among cirrhotic patients in the US, reflecting the rarity of this complication. There have been no reports on the annual incidence of NF in the cirrhotic population. However, in a recent population-based cohort study in Taiwan by Hung and colleagues, the investigators reported development NF in 0.7% of previously hospitalized cirrhotic patients, followed for 3 years [9]. The investigators also noted nearly two-fold higher odds for development of NF in cirrhotic vs. non-cirrhotic patients [9], reflecting the higher overall risk of infectious complications among the former. We found that the progressively increasing annual volume of NF associated with cirrhosis noted in the present cohort was matching a corresponding demand for hospitalization among cirrhotic patients in the state, the latter finding being in line with reported national trends [24]. However, the incidence of NF among the cirrhotic population in the US remains unknown.

The predominance of males and Hispanic ethnicity in the present cohort is similar to findings among NF patients in the state [8], and nationally for the former [25]. In addition, males and Hispanics are overrepresented among cirrhosis patients [24]. Elderly NF hospitalizations were a minority in the present cohort, in line with both findings in NF in the general population [8] and among cirrhosis patients [24]. The source of the observed downtrend in elderly NF hospitalizations, similar to NF in the general population [8], remains unclear.

The majority of NF hospitalizations associated with cirrhosis had non-cirrhosis chronic comorbidities, with diabetes mellitus being the most common reported condition. These findings are similar to previous reports on NF in cirrhosis [15, 16] and those on NF in the general population [8], with diabetes being a well-recognized additional risk factor for NF [26]. We found a significant rise in the overall burden of chronic illness in the present cohort, as reflected by increasing Deyo-Charlson scores. Similar trends were noted among NF patients in longitudinal studies in the general population [8]. However, the sources of the observed trends are unclear and warrant further study in other populations.

An additional site of infection was reported in nearly one in three NF hospitalizations, mostly involving the urinary and intra-abdominal sites. It is unclear whether reported infections preceded, followed, or occurred synchronously with NF. There have been no reports, to our knowledge, on non-NF sites of infection among NF patients with cirrhosis. We previously reported occurrence of an additional site of infection in 34% of NF hospitalizations in the general population [8], and Widjaja and colleagues described infections of other sites, complicating the course of NF in 76% of an Australian cohort [22]. Urinary tract infections were among the most common sites in both latter studies. However, the limited data on non-NF infections preclude assessment of their impact on the outcomes of NF patients.

The majority of NF hospitalizations required care in the ICU, at a rate markedly higher than that reported in the general NF population [8], likely related to the higher severity of illness, as reflected by higher rate of OF among the former. There have no prior reports of ICU utilization among NF patients with cirrhosis.

Nearly three in four NF hospitalizations had one or more OF, markedly higher than a 30.7% [27] to 40% rate [8] among NF patients in the general population. Previous case series of NF associated with cirrhosis did not describe organ failure patterns. The predominance of hematological failure in the present cohort can be expected given a prevalent coagulation dysfunction in among cirrhotic patients, and which can further deteriorate during acute infectious illness. When reported in the general population with NF, renal failure was the second most common affected organ system, similar to the present findings [8, 28]. An increased risk of organ failure among infected cirrhotic patients has been reported by Foreman and colleagues [1] and likely accounts in part for the worse outcomes in this population. Our findings of rising rates of all individual organ failures likely reflect actual increase in severity of illness, rather than up-coding by clinicians. This hypothesis is supported by the unchanged rates of use of related life-support interventions among NF hospitalizations with corresponding organ failure and the over 2.5-fold rise in rates of discharge to long-term care facilities. It is likely that increasing burden of non-cirrhosis chronic comorbidities over time in our cohort has contributed to higher risk of OF, similar to prior reports on severe sepsis [29]. However, we cannot exclude the role of possible increased virulence and drug resistance of infecting pathogens. Further studies are required to examine the sources of rising rates of organ failure among NF cirrhotic patients and to corroborate our findings.

Amputation was rarely used in the present cohort, lower than the reported rates between 4.8% [16] and 29.4% [15] among NF patients with cirrhosis in Taiwan. However, in addition to differences of population mix and practice setting, the latter studies involved very small sample size (42 [16] and 17 [15] patients, respectively). The amputation rate among NF patients in the general population ranged from 3.9% [8] to 5.4% [7] in recent population-based studies. Thus, most contemporary NF patients can be managed without need for amputation. Hyperbaric oxygen therapy (HBO) was rarely used in the present cohort, similar to a recent population-based study of NF in the general population in Texas [8]. However, the role of HBO in NF patients remains uncertain [26].

NF patients with cirrhosis had prolonged hospitalization, shorter than those reported in Taiwan which ranged from about 20 [16] to 23.9 days [15], likely related to differing population mix and practice patterns. The fiscal burden of NF associated with cirrhosis has not been previously reported, and was markedly higher than those for NF hospitalizations in the general population [8], likely due to the higher severity of illness and associated increased use of critical care resources among the former, with NF in the present cohort exceeding charges for the most expensive hospital condition in the state [30]. The lack of significant change in hospital length of stay and hospital charges occurred despite the noted rise in severity of illness and increased use of life-support interventions. These apparent contradictory trends may reflect improved care efficiencies, but more likely due to possible earlier discharge to long-term care facilities, which rose more than 2.5-fold during study period.

Hospital mortality occurred in nearly one in three NF hospitalizations with cirrhosis, being nearly three-fold higher than that described in contemporary reports on NF in the general population [6-8], in line with the higher risk of death among infected cirrhosis patients, as compared to non-cirrhotic population [1]. Case fatality among NF patients with cirrhosis ranged from 33.3% [16] to 64.7% [15] in two small cohort studies from Taiwan.

There was a nonsignificant upward trend of hospital mortality, likely due to the concomitant marked increase in severity of illness, as reflected by increasing number of OFs. It is conceivable that the lack of more significant increase in hospital mortality in the present cohort rather underestimates trends of short-term mortality due to change in location of patients’ death. This hypothesis is supported by the more than doubling rate of discharges to long-term care facilities among hospital survivors, indicating, as noted earlier, higher residual morbidity. Indeed, a recent report by Reineck and colleagues documented that a marked variability in adjusted hospital mortality is a function of a “discharge bias” related to varying discharge practices [31], corroborating an earlier study by Hall et al who noted that transfers to long-term acute care facilities can explain a significant proportion of the variation of reported hospital mortality [32]. Although marked decrease in hospital mortality among patients with cirrhosis in the US over the past decade has been recently reported by Schmidt and colleagues [24], the overall mortality rate among patients with cirrhosis was trending upwards over the same period [33]. However, Schmidt et al did not report on non-hospice hospital disposition among survivors [24].

Our findings should be considered in the context of several limitations. First, a retrospective design and use of an administrative data set with their attendant limitations affect the interpretation of our results. However, the rarity of NF, especially among patients with cirrhosis, limits population-level approaches to study this condition. In addition, the de-identified data do not allow accounting for multiple hospitalizations by the same patient during specific period. However, similar approach with the aforementioned limitations was used by other investigators of NF [6, 7].

We have identified hospitalizations with liver cirrhosis based on specific ICD-9-CM codes. Thus, we cannot exclude possible misclassification. However, our approach is similar to that reported by other investigators involving population studies [16, 24, 34].

The case definition of NF in the present study has been based on ICD-9-CM coding at reporting hospitals. Administrative data sets do not provide information on pathological confirmation of NF diagnosis, raising a potential for misclassification. Nevertheless, NF diagnoses were reported very sparingly (0.1%) among cirrhotic hospitalizations in our cohort and it is unlikely that miscoding occurred systematically or incrementally over time and thus misclassification is unlikely to explain the rise in the annual volume of NF hospitalizations. On the other hand, we cannot exclude underestimation of NF in our cohort. Finally, our case identification approach is similar to prior population-level reports of NF [6].

The use of administrative data in our study precluded access to information on sites of infection, the timeliness of diagnosis of NF, and to the details, time course, and appropriateness of antimicrobial therapy, surgical source control, and resuscitative interventions, all of which may vary across institutions and individual clinicians, and likely have affected the observed resource utilization and outcomes. However, as noted earlier, similar constraints affect interpretation of prior population-level studies of NF [6, 7]. Finally, because the state of Texas does not provide tools to convert hospital charges to costs, we reported hospital charges rather than costs of care, limiting comparisons with other cost data. However, the available charge data allowed comparisons within state population.

### Conclusion

We report the first longitudinal population-level study of NF among patients with cirrhosis. The annual incidence of NF among hospitalized cirrhotic patients remained unchanged, while the burden of chronic comorbidities and severity of illness among the former rose markedly over the past decade. The majority of NF hospitalizations required admission to an ICU, with common use of life-support interventions. NF patients with cirrhosis required prolonged hospitalization with high hospital charges, making NF possibly the costliest hospital diagnosis in the state. Hospital mortality was high and tended to rise, but may have underestimated short-term mortality, while NF was likely associated with substantial residual morbidity among hospital survivors. Further studies of NF in patients with cirrhosis are warranted in other populations to corroborate our findings.
